# Oxeiptosis-Associated Molecular Subtyping and Immune Microenvironment Heterogeneity in Osteoarthritis

**DOI:** 10.3390/biomedicines14092079

**Published:** 2026-09-16

**Authors:** Huiwen Huang, Xuwu Chen, Wenxia Sun, Xiyan Lan, Zhiyi Zeng, Yushang Liu, An Yin, Qiong Deng, Yanbiao Zhong, Maoyuan Wang

**Affiliations:** 1 School of Rehabilitation, Gannan Medical University, Ganzhou 341000, China; m18470741614@163.com (H.H.); 3273949852chen@gmail.com (X.C.);; 2 Department of Rehabilitation Medicine, The First Affiliated Hospital of Gannan Medical University, Ganzhou 341000, China; 3 Jiangxi Medical College, Nanchang University, Nanchang 330031, China; 4 First Clinical Medical College, Gannan Medical University, Ganzhou 341000, China; 5 Ganzhou Key Laboratory of Rehabilitation Medicine, Ganzhou 341000, China; 6 Ganzhou Intelligent Rehabilitation Technology Innovation Center, Ganzhou 341000, China

**Keywords:** osteoarthritis, oxeiptosis, molecular subtypes, heterogeneity, immunity

## Abstract

**Background:** Oxeiptosis is a ROS-induced, caspase-independent form of regulated cell death, but its role in osteoarthritis (OA) remains largely unexplored. This study aimed to identify oxeiptosis-associated molecular markers in OA synovium and establish an oxeiptosis-based molecular classification system. **Methods:** This study integrated five publicly available GEO datasets for comprehensive analysis. GSE55235 and GSE55457 were used as discovery cohorts to identify DEGs between OA and normal synovial tissues. The GSE206848 dataset was utilized to perform correlation analysis with the key oxeiptosis regulators, including *KEAP1*, *PGAM5*, *AIFM1*, and *CUL3*, thereby establishing an oxeiptosis-associated gene set. These genes were intersected with OA-related DEGs to obtain ORDEGs. Subsequently, LASSO regression, SVM-RFE, and RF algorithms were jointly applied to identify hub differential genes. Based on the identified oxeiptosis-related feature genes, molecular subtypes of OA were constructed, with the GSE46750 dataset used for machine learning-based feature selection. Consensus clustering was then performed based on the selected features to identify distinct OA molecular subtypes. The immune microenvironment characteristics and potential regulatory networks of different subtypes were further investigated using CIBERSORT, ESTIMATE, and WGCNA. Finally, an independent GSE89408 cohort was employed for external validation. **Results:** A total of 159 common differentially expressed genes were identified from the two OA synovial cohorts, which were mainly enriched in cellular response to hydrogen peroxide, the MAPK signaling pathway, the PI3K-Akt signaling pathway, and NF-κB-related inflammatory processes. Further screening identified seven ORDEGs, including *OTUD4*, *GATM*, *KTN1*, *FGGY*, *ACACB*, *RERE*, and *APLP2*. Machine learning analysis ultimately identified *ACACB* and *FGGY* as potential molecular features of OA. Molecular clustering based on oxeiptosis-related features demonstrated that OA samples could be stably classified into two subtypes, C1 and C2. The C2 subtype exhibited higher ORDEG scores, increased *ACACB* expression levels, greater M1 macrophage infiltration, and higher ESTIMATE scores, indicating oxidative stress-related transcriptional characteristics and enhanced inflammatory features, whereas *FGGY* was mainly highly expressed in the C1 subtype. WGCNA further revealed that the salmon module closely associated with the C2 subtype was mainly enriched in calcium signaling, focal adhesion, cytoskeletal remodeling, and cell adhesion-related pathways. In the independent GSE89408 validation cohort, exploratory clustering analysis again identified two potential molecular subtypes, and *FGGY* remained significantly differentially expressed between the two subtypes. In addition, *ACACB* and *FGGY* showed AUC values of 0.735 and 0.647, respectively, for distinguishing OA from normal synovial tissues. **Conclusion:** This study establishes an oxeiptosis-associated molecular subtyping framework for OA synovium and identifies *ACACB* and *FGGY* as candidate oxeiptosis-associated genes. Further analyses revealed distinct immune microenvironment characteristics and transcriptional regulatory patterns associated with different oxeiptosis states. External validation provided partial support for the reproducibility of these molecular patterns, particularly the *FGGY*-related features. These findings provide transcriptomic evidence for exploring molecular heterogeneity in OA and generate hypotheses for future mechanistic and clinical validation studies.

## 1. Introduction

Osteoarthritis (OA) is one of the most prevalent degenerative joint diseases worldwide and is characterized by progressive joint dysfunction. OA can be broadly classified into primary and secondary forms, with its major pathological features including cartilage degeneration, osteophyte formation, and synovial inflammation [1]. Epidemiological studies indicate that OA affects more than 528 million individuals worldwide, representing approximately 7% of the global population, and ranks among the leading causes of years lived with disability (YLDs) [2]. Current projections indicate that the global prevalence of OA is expected to continue rising through 2040, with the total number of cases increasing from 19.4 million to 38.8 million [3]. In the context of global population aging, the socioeconomic and clinical burden of OA is expected to further escalate. It is well established that synovitis typically precedes cartilage degeneration, and synovial inflammation is a key pathological feature in the progression of OA [4,5]. Among the various pathogenic factors contributing to synovitis, oxidative stress, characterized by excessive accumulation of reactive oxygen species (ROS), plays a pivotal role [6,7]. However, when ROS levels exceed the body’s antioxidant capacity, pathological oxidative stress is induced, resulting in tissue structural damage [8]. Notably, recent studies have revealed that ROS not only act as mediators of oxidative damage but also serve as important signaling molecules involved in cellular regulation. ROS can influence the tissue microenvironment by inducing various forms of regulated cell death (RCD) [9]. Among these, ROS-related RCD forms, including ferroptosis, parthanatos, and oxeiptosis, have been shown to contribute to chondrocyte dysfunction and the pathological progression of OA [10]. However, current research on RCD in OA has primarily focused on classical death pathways such as ferroptosis, with emphasis on mechanisms including iron metabolism disorders, lipid peroxidation, and inflammatory responses. In contrast, the role of the oxeiptosis pathway, which is centered on the regulation of ROS and oxidative stress, in OA progression remains under-explored.

Oxeiptosis is a newly discovered form of ROS-induced, caspase-independent regulated cell death, first reported by Holze et al. in 2018 [11]. This process is mediated by the *KEAP1–PGAM5–AIFM1* signaling axis. Upon detection of elevated ROS levels, the redox sensor *KEAP1* releases the phosphatase *PGAM5*, which subsequently mediates the dephosphorylation of *AIFM1* at serine residue 116, initiating oxeiptosis-associated cell death [12]. Increasing evidence has demonstrated that oxeiptosis is involved in the progression of various oxidative stress-related diseases. For example, in models of oxidative stress and Alzheimer’s disease, *KEAP1*, *PGAM5*, and *AIFM1* are significantly upregulated, while knockdown of these genes improves cell survival under oxidative stress conditions [13]. Meanwhile, accumulating studies have confirmed that oxeiptosis contributes to redox homeostasis imbalance during the progression of infections, immune-mediated diseases, tissue degeneration, lung injury, and tumors, thereby regulating disease progression and prognosis [14]. Given that excessive ROS accumulation represents a key pathological feature of OA, and that oxeiptosis, as a specific ROS-dependent form of RCD, may theoretically participate in OA-related cellular homeostasis imbalance, recent reviews have proposed oxeiptosis as a potential mechanism involved in chondrocyte death in OA. In addition, the key signaling molecules *PGAM5* and *AIFM1* have been identified as potential targets for inhibiting chondrocyte death [10]. However, current research on oxeiptosis in OA remains at an early exploratory stage, with a particular lack of systematic investigations into synovial tissue and its inflammatory microenvironment.

As a complex degenerative disease characterized by significant molecular and cellular heterogeneity, OA may exhibit varying states of oxidative stress and patterns of cell death across different patients and disease stages. Therefore, elucidating the molecular characteristics associated with oxeiptosis and its potential molecular subtypes is of great significance for understanding the heterogeneity of OA and identifying new therapeutic targets. This study aimed to initially explore the expression patterns of potential oxeiptosis-associated candidate genes during the development of OA and to identify potential key associated genes and molecular heterogeneity through machine learning, immune infiltration analysis, WGCNA analysis, and external validation.

## 2. Materials and Methods

### 2.1. Overview of the Study Population

This study systematically searched the Gene Expression Omnibus (GEO) database for human OA synovial tissue datasets and included five publicly available gene expression datasets (GSE55235, GSE55457, GSE206848, GSE46750, and GSE89408) [15]. These datasets were utilized according to their respective research objectives, including DEG discovery, oxeiptosis-associated correlation screening, machine learning-based feature selection, molecular subtype construction, and independent external validation (Table 1).

**Figure 1:**
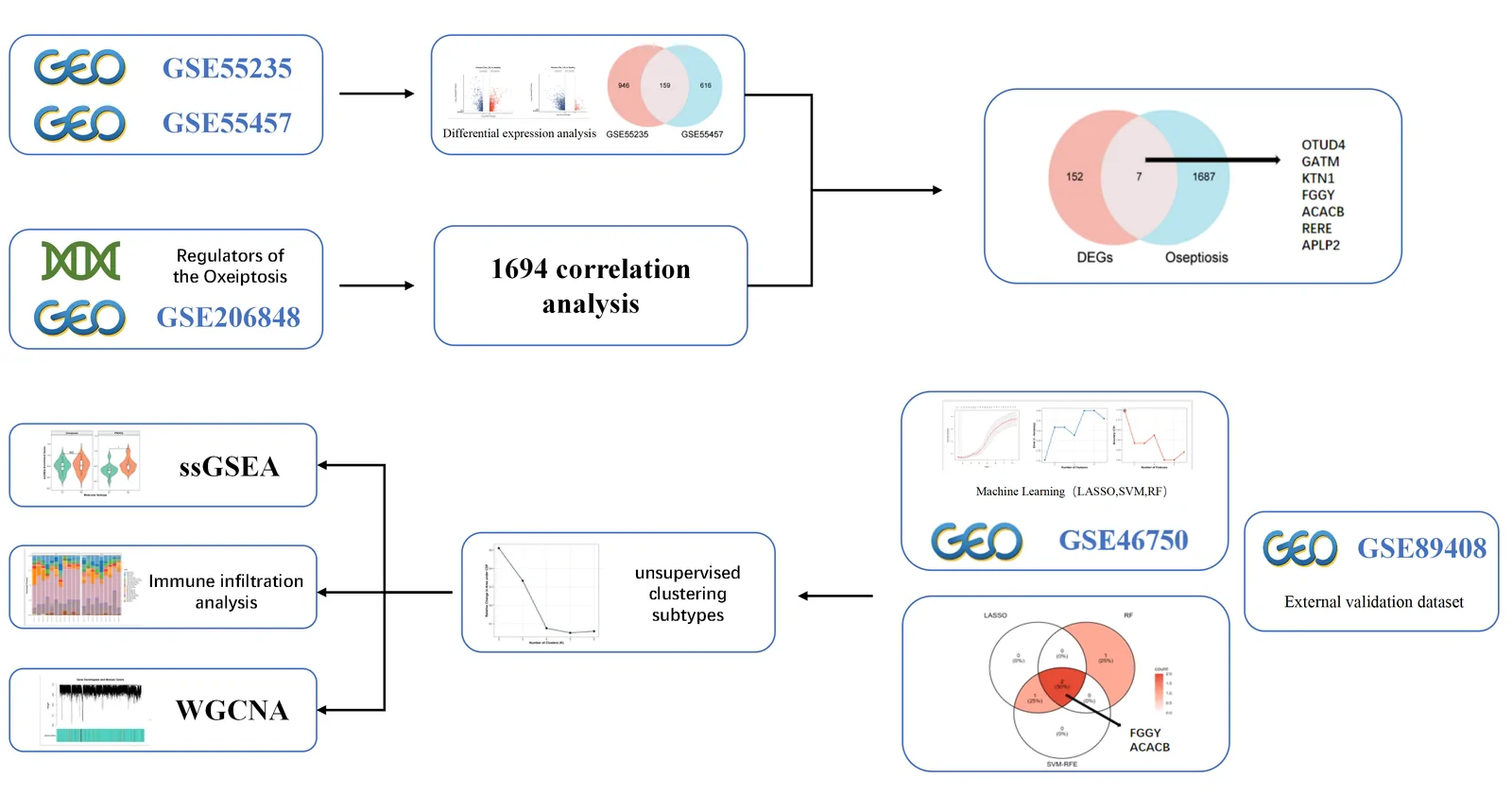
Schematic overview of the study design and bioinformatics workflow. This workflow summarizes the integrated analysis strategy for identifying oxeiptosis-related molecular features in OA. Transcriptomic datasets from GEO were analyzed to identify OA-related differentially expressed genes and genes associated with the oxeiptosis-related expression signature. Machine learning algorithms were applied to screen key candidate genes, followed by molecular clustering based on *ACACB* and *FGGY* expression profiles. The identified molecular groups were characterized through immune infiltration analysis, ssGSEA, and WGCNA. An independent GEO cohort (GSE89408) was used for external validation of the identified molecular features and clustering patterns. OA, osteoarthritis; GEO, Gene Expression Omnibus; ssGSEA, single-sample gene set enrichment analysis; WGCNA, Weighted Gene Co-expression Network Analysis.

GSE55235 and GSE55457 were used to identify OA-related differentially expressed genes (DEGs). Each dataset included 10 OA samples and 10 healthy control samples. Differential expression analyses were performed separately for the two datasets, and the overlapping DEGs were subsequently identified. GSE206848 was used to screen candidate genes correlated with oxeiptosis-associated expression changes under OA pathological conditions. This dataset included 7 OA synovial samples, 7 normal synovial tissue samples, and 2 rheumatoid arthritis (RA) synovial samples. Correlation analysis was performed only using OA and normal synovial tissue samples, while RA samples were excluded from this analysis. GSE46750 was used as the training set for machine learning-based feature selection to identify potential key genes. This dataset consisted of 24 paired synovial biopsy samples from 12 OA patients, including 12 samples from inflammatory regions and 12 samples from non-inflammatory regions. As both sample groups were derived from OA patients, this dataset was used to identify candidate features associated with local inflammatory phenotypes in OA synovium rather than as an independent diagnostic validation cohort comparing OA patients with healthy controls. Molecular subtype analysis was performed using the batch-effect-corrected and integrated GSE55235 and GSE55457 datasets, including only the 20 OA samples without healthy control samples. GSE89408 served as an independent validation cohort, consisting of 22 OA samples and 28 healthy control samples. This dataset was not involved in DEG screening, correlation filtering, or machine learning-based feature selection. All 50 samples were used to evaluate the external discriminatory performance of *ACACB* and *FGGY* in distinguishing OA from healthy controls. The overall workflow is shown in Figure 1.

### 2.2. Identification of ORDEGs (Oxeiptosis-Related Differentially Expressed Genes)

Differential expression analysis was performed separately in the GSE55235 and GSE55457 cohorts using the “limma” R package. Genes with |log FC| > 1 and Benjamini–Hochberg adjusted false discovery rate (BH-FDR) < 0.05 were identified as differentially expressed genes (DEGs) between OA patients and normal synovial tissues. Multiple hypothesis testing was corrected using the Benjamini–Hochberg method, and common DEGs with consistent expression directions between GSE55235 and GSE55457 were subsequently selected as OA-related DEGs. According to previous studies, *KEAP1*, *PGAM5*, and *AIFM1* are considered key regulatory molecules in the classical oxeiptosis signaling axis. In addition, *CUL3*, as an important upstream regulator involved in *KEAP1*-mediated oxidative stress regulation, has also been incorporated into oxeiptosis-related molecular feature construction in recent bioinformatics studies. Therefore, based on previous studies and published oxeiptosis-related molecular analyses, *KEAP1*, *PGAM5*, *AIFM1*, and *CUL3* were selected as oxeiptosis-associated regulatory factors [11,16,17]. In the GSE206848 cohort, pairwise Pearson correlation analyses were performed between genes in the dataset and these four oxeiptosis-related regulatory genes. Genes showing strong correlations with any of the marker factors were considered potential oxeiptosis-related candidate genes (ORGs), defined as genes with Pearson correlation coefficients |r| > 0.5 and BH-FDR < 0.05. To evaluate the robustness of the correlation threshold selection, sensitivity analyses were performed using alternative Pearson correlation thresholds of |r| > 0.4 and |r| > 0.6, while maintaining the same BH-adjusted significance criterion (FDR < 0.05). (Supplementary Figure S2). For genes exhibiting stronger correlations, Pearson correlation coefficients and 95% confidence intervals calculated using Fisher’s r-to-z transformation were additionally reported. The intersection between ORGs and DEGs was defined as oxeiptosis-related differentially expressed genes (ORDEGs), which were subsequently used as input features for machine learning model construction.

### 2.3. Machine Learning Prediction of Key ORDEGs

The GSE46750 dataset was used for machine learning-based selection of ORDEGs. This dataset included 24 paired synovial tissue samples from 12 OA patients, with each patient providing one sample from an inflammatory region and one sample from a normal/reactive region. Probe IDs were converted into gene symbols using the illuminaHumanv4.db package, and when multiple probes corresponded to the same gene, the average expression value was calculated. Before model training, gene expression values were Z-score normalized within each training fold, and the normalization parameters derived from the training set were applied to the corresponding validation set. Three machine learning algorithms, including LASSO, SVM-RFE, and random forest, were applied for feature selection. To preserve the paired structure of the data, all cross-validation procedures and repeated resampling were performed at the patient level, ensuring that paired samples from the same patient were simultaneously assigned to either the training or validation set. Model performance was evaluated using patient-level leave-one-patient-out cross-validation (LOPO-CV), in which both paired tissue samples from one patient were retained as the validation set each time, while samples from the remaining 11 patients were used for model training, resulting in 12 outer validation iterations. Within the training set, patient-stratified five-fold cross-validation was performed for parameter optimization. LASSO analysis was performed using the glmnet package to construct a binomial regression model with alpha = 1, and lambda.min was selected based on cross-validation error. SVM-RFE was performed using the e1071 package. Random forest models were constructed using the randomForest package, with the final prediction model consisting of 1000 decision trees, and the optimal parameter was selected from a range of 1–7.

To evaluate feature selection stability, 200 rounds of patient-level repeated subsampling were performed. In each iteration, 9 of the 12 patients were randomly selected without replacement, while retaining all paired tissue samples from the selected patients. LASSO, SVM-RFE, and random forest feature selection were repeated in each resampling iteration, with patient-stratified three-fold cross-validation applied internally. For random forest stability analysis, 500 decision trees were used in each iteration, and genes were ranked according to Mean Decrease Gini. The selection frequency of each gene across 200 resampling iterations was calculated. The top three genes ranked by selection frequency from each algorithm were extracted, and their intersection was defined as the final candidate genes. ROC curves were generated using the pROC package based on the outer predictions from patient-level LOPO-CV, and the corresponding AUC values were calculated. The 95% confidence intervals of AUC were estimated using 2000 patient-cluster bootstrap iterations, with both paired tissue samples from each patient sampled together in each iteration. To evaluate the external diagnostic performance of candidate biomarkers, an independent validation was conducted in the GSE89408 cohort, which was not involved in DEG screening, correlation filtering, machine learning-based feature selection, model construction, or threshold determination. This cohort included 22 OA samples and 28 healthy control samples. The AUC values and corresponding 95% confidence intervals of the core genes were calculated using the pROC package.

### 2.4. Subtype Identification and Functional Scoring

After integrating the GSE55235 and GSE55457 datasets, only the 20 OA samples were included for molecular subtype identification. Consensus k-means clustering was performed based on the standardized expression patterns of the core genes selected by machine learning. Using k-means as the basic clustering algorithm for each resampling iteration, consensus clustering was performed with candidate cluster numbers ranging from k = 2 to 6. For each candidate k value, 1000 resampling iterations were conducted, with 80% of samples randomly selected without replacement in each iteration. The k-means algorithm was implemented using the Hartigan–Wong method, with 20 random initial cluster centers (nstart = 20) and a maximum of 100 iterations (iter.max = 100). A consensus matrix was generated based on the frequency with which samples were assigned to the same cluster across repeated resampling. The optimal number of clusters was determined by evaluating the consensus cumulative distribution function (CDF) curves, relative changes in the area under the CDF curve, and the average within-cluster consensus scores across different k values. CDF was used as an evaluation metric for clustering stability rather than an independent clustering algorithm. After determining the optimal number of clusters, final subtype assignments were obtained using Ward.D2 hierarchical clustering with 1 minus the consensus matrix as the distance measure. Clustering stability and uncertainty were evaluated using silhouette coefficients and within-cluster consensus scores, and consensus matrix heatmaps were generated to visualize the shared clustering patterns among samples. Nearest template prediction (NTP) was applied as a secondary internal consistency assessment method. NTP used the same samples and gene features as consensus clustering, and the concordance between NTP classification and consensus clustering classification was evaluated using confusion matrices, concordance rates, and Spearman correlation coefficients. Since NTP and consensus clustering were based on the same dataset, NTP analysis was used only to assess internal consistency between different classification strategies and was not considered an independent external validation. The stability of subtype classification was further assessed using silhouette analysis, and gene expression patterns among different clusters were visualized using heatmaps. To further evaluate the reliability of subtype assignment, NTP was applied, and the consistency between k-means classification and NTP classification was assessed using confusion matrices and Spearman correlation analysis. In addition, single-sample gene set enrichment analysis (ssGSEA) implemented in the “GSVA” R package was used to calculate oxeiptosis-related functional scores and ORDEG scores for each sample. Differences in enrichment scores between sample clusters were assessed using two-sided Wilcoxon rank-sum tests, and multiple comparisons within the same analysis were corrected using the Benjamini–Hochberg method. The ORDEG score was calculated using ssGSEA based on seven putative oxeiptosis-associated differentially expressed genes (*OTUD4*, *GATM*, *KTN1*, *FGGY*, *ACACB*, *RERE*, and *APLP2*).

To explore the applicability of the same two-gene clustering framework in another OA synovial cohort, exploratory repeated clustering was performed in the 22 OA samples from GSE89408. The analysis used the same *ACACB* and *FGGY* gene features, candidate cluster number range (k = 2–6), number of resampling iterations (1000), sample proportion (80%), and k-means parameters as those used in the discovery cohort. Gene expression values were standardized within the GSE89408 cohort, and the clustering structure of this cohort was determined independently. Internal clustering stability was evaluated using the consensus matrix, CDF curves, relative changes in the area under the CDF curve, average within-cluster consensus scores, and sample-level silhouette coefficients. The resulting sample clusters were descriptively labeled according to the relative expression patterns of *ACACB* and *FGGY*, and two-sided Wilcoxon rank-sum tests were used to compare oxeiptosis and ORDEG scores between clusters, with the corresponding *p* values adjusted using the Benjamini–Hochberg method.

### 2.5. Analysis of Immune Infiltration Among Different Subtypes

To systematically analyze differences in the immune microenvironment between the C1 and C2 subtypes, the expression matrices of GSE55235 and GSE55457 were consistently processed using the same procedures, including non-log2 transformation, gene symbol annotation, and low-quality gene filtering. Subsequently, the CIBERSORT algorithm combined with the LM22 signature matrix was applied with 1000 permutations to quantify the abundance of 22 types of immune cells, retaining only samples with *p* < 0.05 to ensure the reliability of the estimates. Subsequently, the Wilcoxon rank-sum test combined with Benjamini–Hochberg correction was used to compare differences in immune cell abundance between the two subtypes. The Stromal Score, Immune Score, and ESTIMATE Score were compared between groups using two-sided Wilcoxon rank-sum tests. *p* values from the three tests were adjusted for multiple comparisons using the Benjamini–Hochberg method. The median, interquartile range, raw *p* values, and BH-adjusted false discovery rate (FDR) were reported for each subtype. Spearman correlation analysis was performed to assess the associations between the selected hub genes and immune cell infiltration levels.

### 2.6. Network and Functional Analysis

Weighted gene co-expression network analysis (WGCNA) was performed to identify major co-expression modules based on the expression differences in subtype-associated candidate genes. Genes were ranked according to their expression variance among OA samples, and the top 5000 genes with the highest variance were selected for network construction. The goodSamplesGenes() function in the WGCNA package was used to assess sample and gene quality, including the detection of missing values and non-finite values in the expression matrix. Potential outlier samples were identified through hierarchical clustering of samples and standardized sample connectivity, with samples showing standardized connectivity below −2 considered potential outliers. The soft-thresholding power was selected based on the scale-free topology fit index and mean connectivity. Gene expression correlations were transformed into an adjacency matrix, followed by calculation of the topological overlap matrix (TOM) and corresponding dissimilarity matrix. Gene clustering trees were constructed using average linkage hierarchical clustering. Initial co-expression modules were identified using the dynamic tree cut algorithm with parameters set as deepSplit = 2, minClusterSize = 30, and pamRespectsDendro = FALSE. Module eigengenes were subsequently calculated, and modules with eigengene dissimilarity below 0.25 were merged, corresponding to an eigengene correlation coefficient greater than 0.75. Genes not assigned to any specific module were classified into the grey module.

Spearman correlation analysis was performed to evaluate the associations between module eigengenes and the C1/C2 molecular subtypes. Multiple testing correction for module–subtype association analyses was performed using the Benjamini–Hochberg method. A two-sided BH-adjusted *p* value (FDR) < 0.05 was considered statistically significant for module–subtype associations. Functional annotation was performed using the DAVID database based on the Gene Ontology (GO) and the Kyoto Encyclopedia of Genes and Genomes (KEGG) [18]. All analyses were performed using R software (version 4.5.2).

## 3. Results

### 3.1. Identification of Differentially Expressed Genes in the Synovial Tissue of Osteoarthritis

**Figure 2:**
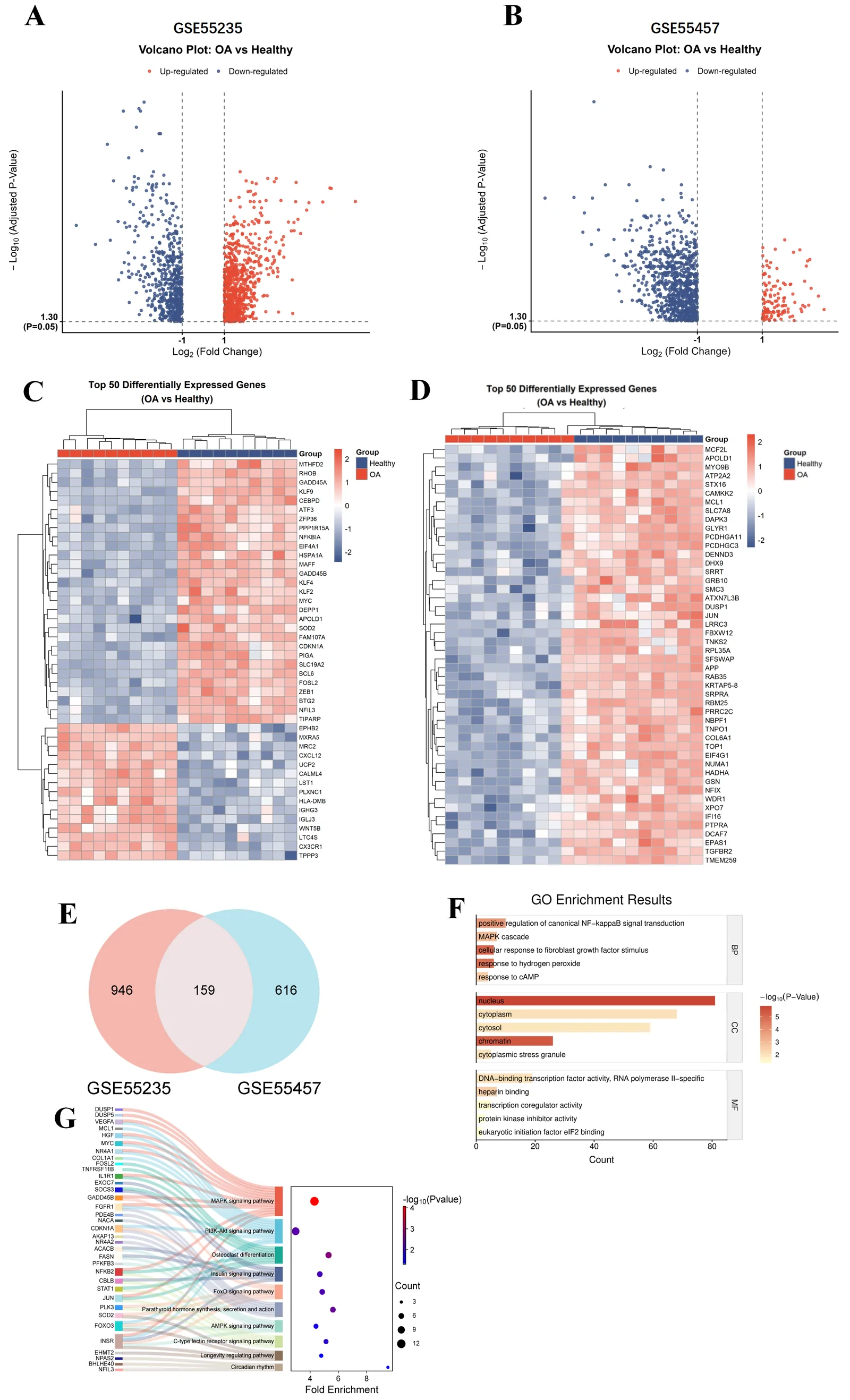
Identification Identification functional enrichment analysis of differentially expressed genes in OA synovium. (**A**,**B**) Volcano plots showing DEGs between OA and normal synovial tissues in the GSE55235 and GSE55457 datasets, respectively. DEGs were identified using the criteria of |log_2_FC| > 1 and BH-FDR < 0.05; (**C**,**D**) Hierarchical clustering heatmaps showing the top 50 upregulated and top 50 downregulated genes with the most significant expression changes in the two datasets, respectively; (**E**) Venn diagram showing the overlapping DEGs between the two datasets, identifying a total of 159 common DEGs; (**F**) GO functional enrichment analysis of the 159 common DEGs; (**G**) KEGG pathway enrichment analysis of the 159 common DEGs. Enrichment analyses were considered statistically significant at BH-FDR < 0.05. OA, osteoarthritis; DEG(s), differentially expressed gene(s); log_2_FC, log_2_ fold change; GO, Gene Ontology; KEGG, Kyoto Encyclopedia of Genes and Genomes.

This study utilized two independent synovial transcriptomic datasets, GSE55235 and GSE55457, to identify DEGs. Differential expression analysis (BH-FDR < 0.05 and |log_2_FC| > 1) revealed widespread transcriptional alterations between OA and healthy synovial tissues in both cohorts (Figure 2A,B). A total of 1105 and 775 DEGs were identified in GSE55235 and GSE55457, respectively. Heatmap analysis based on the top 50 upregulated and downregulated genes in each dataset showed distinct expression patterns between OA and control samples (Figure 2C,D). Intersection analysis of the two DEG sets identified 159 common differentially expressed genes (Figure 2E).

GO and KEGG enrichment analyses were subsequently performed based on the 159 DEGs. KEGG pathway analysis demonstrated that these DEGs were significantly enriched in several classical pathways, including MAPK signaling, PI3K-Akt signaling, FoxO signaling, and osteoclast differentiation. Among these pathways, the MAPK signaling pathway contained the largest number of enriched genes and exhibited the strongest enrichment significance (Figure 2G). GO functional enrichment analysis revealed significant enrichment of biological processes, including positive regulation of classical NF-κB signaling, MAPK cascade, cellular response to fibroblast growth factor stimulus, and cellular response to hydrogen peroxide. In addition, enriched cellular components included cytoplasmic stress granules, while enriched molecular functions included DNA-binding transcription factor activity (Figure 2F). The enrichment of biological processes associated with cellular responses to hydrogen peroxide indicates the involvement of oxidative stress-related processes in OA synovial tissue and provides a basis for further investigating the potential role of oxidative stress-dependent programmed cell death (oxeiptosis) in OA.

### 3.2. Functional Enrichment and Identification of Differentially Expressed Genes Associated with Oxeiptosis

**Figure 3:**
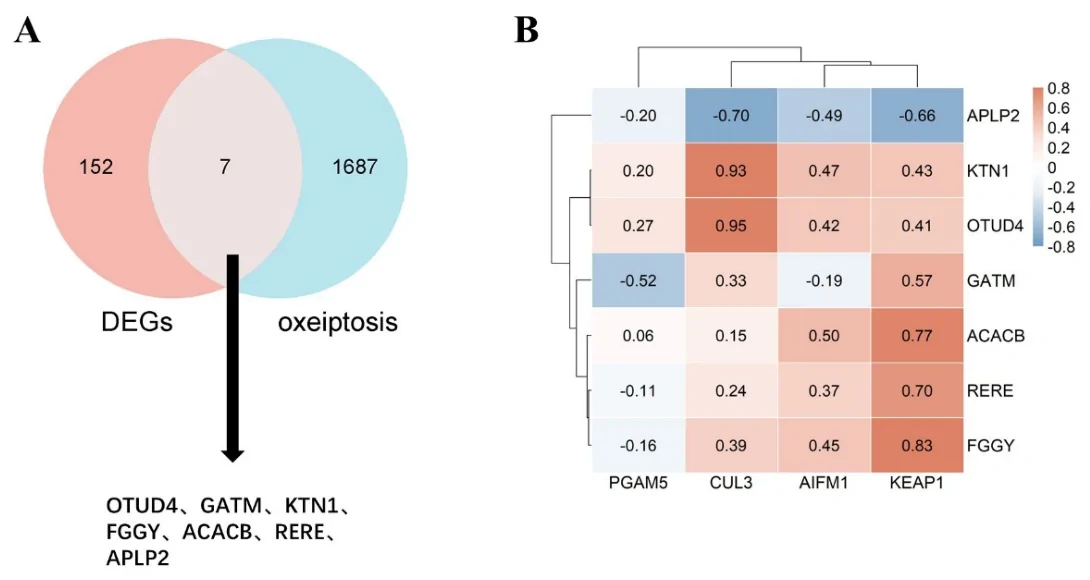
Identification of oxeiptosis-related differentially expressed genes and correlation analysis. (**A**) Venn diagram showing the intersection of DEGs and the oxeiptosis-related gene set, identifying seven potential oxeiptosis-related candidate genes; (**B**) Correlation heatmap showing the associations between the seven candidate genes and core oxeiptosis regulatory factors. DEG(s), differentially expressed gene(s).

By integrating the oxeiptosis-related regulatory factors (*KEAP1*, *PGAM5*, *AIFM1*, and *CUL3*) and performing correlation analysis in the GSE206848 dataset, oxeiptosis-related genes were identified, resulting in a set of 1694 ORGs. By intersecting the 159 DEGs with the 1694 ORGs, seven ORDEGs were identified, including *OTUD4*, *GATM*, *KTN1*, *FGGY*, *ACACB*, *RERE*, and *APLP2* (Figure 3A). Correlation analysis with oxeiptosis regulatory factors revealed significant associations between these seven genes and the identified regulatory factors (Figure 3B), suggesting that these candidate genes may be associated with oxeiptosis-related molecular characteristics.

### 3.3. Machine Learning Identified ACACB and FGGY as Candidate Oxeiptosis-Related Molecular Features in Osteoarthritis

**Figure 4:**
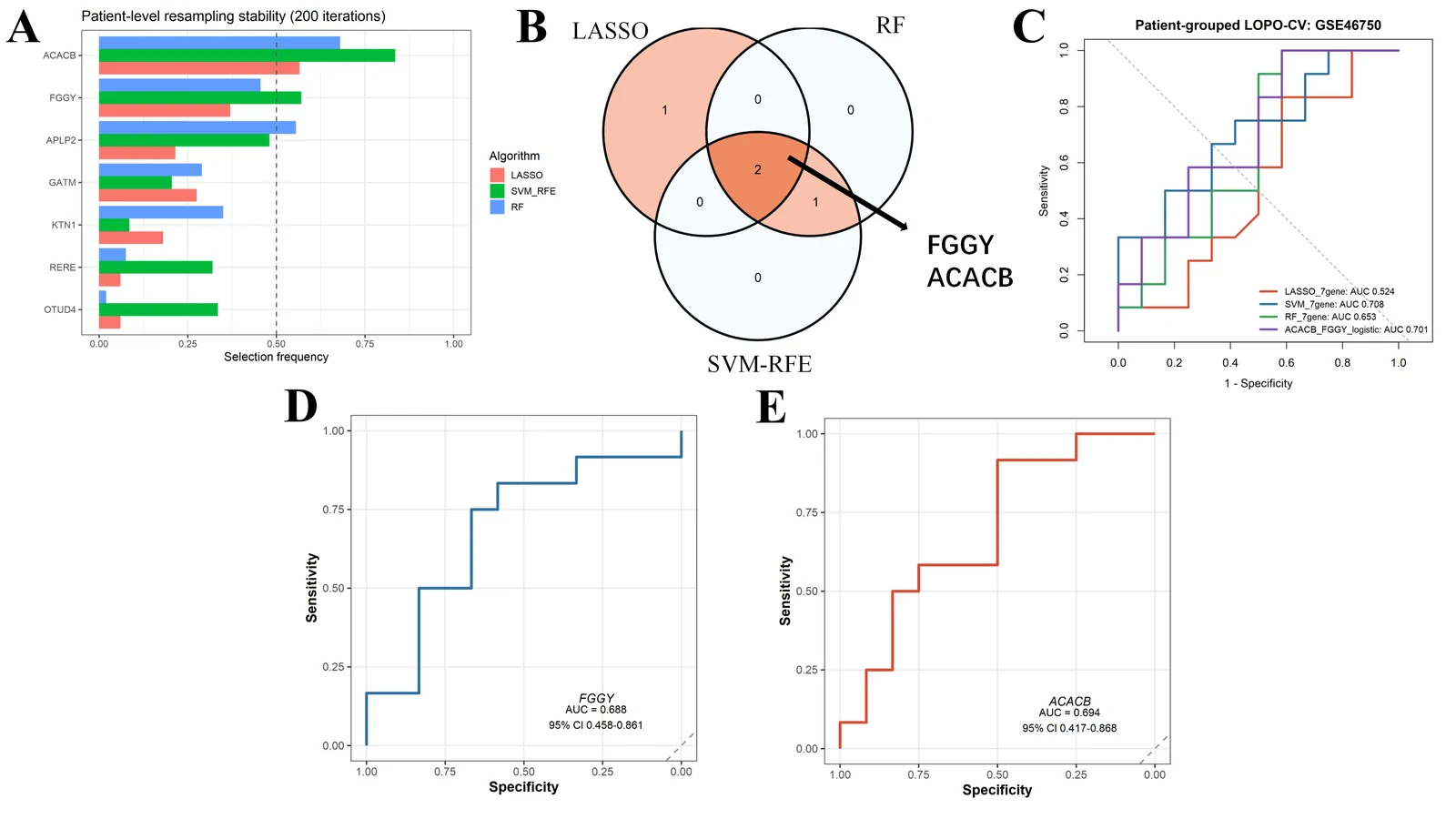
Identification of key candidate genes associated with the oxeiptosis-related expression signature using machine learning approaches. (**A**) Patient-level resampling stability analysis (200 iterations) showing the selection frequencies of seven putative oxeiptosis-related differentially expressed genes across three machine learning algorithms, including LASSO, SVM-RFE, and RF; (**B**) Venn diagram showing the overlap of candidate genes identified by the LASSO, SVM-RFE, and RF algorithms; (**C**) ROC curves of different machine learning models evaluated using patient-level LOPO-CV in the GSE46750 dataset. The classification performances of seven-gene models constructed by LASSO, SVM-RFE, and RF, as well as the two-gene *ACACB*-*FGGY* logistic regression model, were assessed; (**D**,**E**) Single-gene ROC curve analyses evaluating the discriminatory performance of *FGGY* (**D**) and *ACACB* (**E**). The AUC values and 95% confidence intervals are shown for each candidate gene. LASSO, least absolute shrinkage and selection operator; SVM-RFE, support vector machine-recursive feature elimination; RF, random forest; ROC, receiver operating characteristic; LOPO-CV, leave-one-patient-out cross-validation; AUC, area under the curve; CI, confidence interval; DEG(s), differentially expressed gene(s).

To further identify reliable candidate genes associated with the oxeiptosis-related expression signature, three independent machine learning algorithms, including LASSO, SVM-RFE, and RF, were applied to seven potential ORDEGs (*OTUD4*, *GATM*, *KTN1*, *FGGY*, *ACACB*, *RERE*, and *APLP2*). First, 200 iterations of patient-level resampling analysis were performed to evaluate the robustness and consistency of candidate gene selection across different machine learning algorithms. The detailed model fitting procedures, parameter optimization curves, and random forest feature importance analyses are presented in Supplementary Figure S1. The selection frequency of each candidate gene in the LASSO, SVM-RFE, and RF models was calculated. Among the seven candidate genes, *ACACB* exhibited a relatively higher selection frequency across all three algorithms, with selection frequencies of 56.5%, 83.5%, and 68.0% in the LASSO, SVM-RFE, and RF models, respectively. *FGGY* also showed stable selection performance, with selection frequencies of 37.0%, 57.0%, and 45.5%, respectively (Figure 4A). The intersection of the top three genes ranked by selection frequency from each algorithm identified *ACACB* and *FGGY* as the final candidate genes. Therefore, *ACACB* and *FGGY* were selected as key candidate genes associated with the oxeiptosis-related expression signature (Figure 4B).

To further evaluate the discriminatory performance of these candidate genes, ROC analyses were performed. In the patient-level LOPO-CV analysis using the GSE46750 dataset, the combined *ACACB*-*FGGY* logistic regression model demonstrated improved classification performance compared with individual machine learning models, with an AUC of 0.701. The individual models showed moderate predictive performance, including LASSO (AUC = 0.524), SVM-RFE (AUC = 0.708), and RF (AUC = 0.653) (Figure 4C). Furthermore, the independent discriminatory ability of the two selected genes was assessed separately. *FGGY* showed an AUC of 0.688 (95% CI: 0.458–0.861), while *ACACB* showed an AUC of 0.694 (95% CI: 0.417–0.868), suggesting that both genes exhibited potential discriminatory ability for distinguishing molecular states associated with the oxeiptosis-related expression signature (Figure 4D,E). Collectively, these findings identified *ACACB* and *FGGY* as robust candidate genes associated with the oxeiptosis-related molecular signature through multiple machine learning approaches. These genes were subsequently used for downstream molecular subtype analysis.

### 3.4. Construction of OA Molecular Subtypes Based on Oxeiptosis

**Figure 5:**
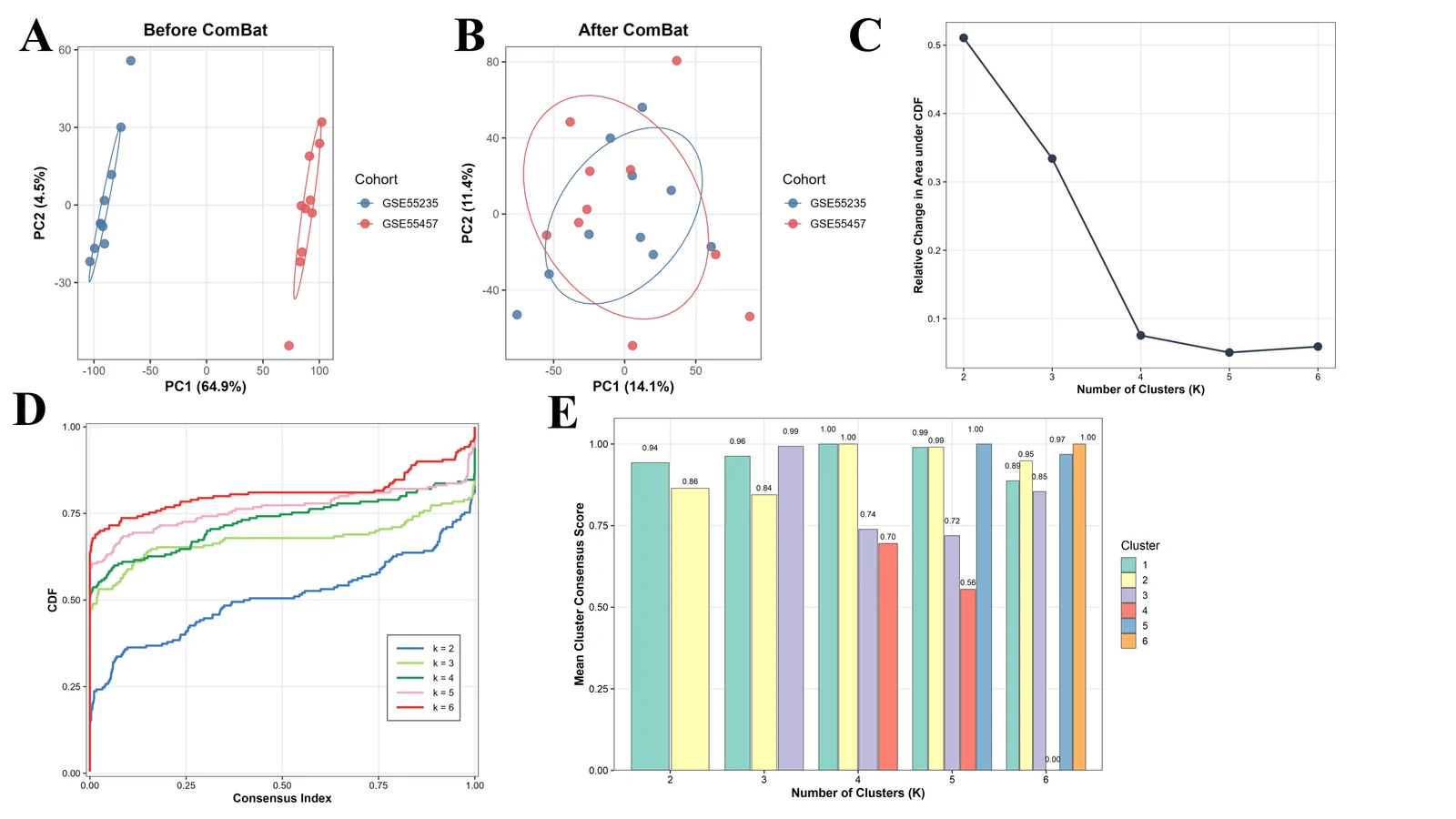
Construction of OA molecular subtypes based on oxeiptosis-related feature genes. (**A**) PCA scatter plot before ComBat batch correction; (**B**) PCA scatter plot after ComBat batch correction; (**C**) Relative changes in the area under the CDF curve for different clustering numbers K; (**D**) Consistency CDF curves for different K values; (**E**) Average consistency scores for each subtype at different K values. OA, osteoarthritis; PCA, principal component analysis; CDF, cumulative distribution function; ComBat, empirical Bayes-based batch effect correction method.

The expression matrices of GSE55235 and GSE55457 were merged, and the ComBat method was applied to reduce batch effects and analyze oxeiptosis-associated molecular heterogeneity in OA. Principal component analysis demonstrated that the dataset-dependent separation observed before batch correction was substantially reduced after correction, indicating successful batch effect correction and data integration (Figure 5A,B). Subsequently, based on oxeiptosis-associated genes and the corrected expression profiles, OA samples were classified into molecular subtypes using a consensus clustering algorithm to determine the optimal number of clusters. The results showed that the relative increase in the area under the CDF curve gradually decreased with increasing cluster numbers (Figure 5C). The CDF curve indicated that clustering at K = 2 showed high stability (Figure 5D). Furthermore, the average consistency scores of the subtypes demonstrated that both subtypes achieved internal consistency scores higher than 0.85 at K = 2, indicating that the clustering results were robust and reliable (Figure 5E). Considering clustering stability and the feasibility of subsequent between-group comparative analyses, K = 2 was ultimately selected as the optimal clustering parameter, dividing OA samples into two molecular subtypes, designated as C1 and C2.

### 3.5. Internal Stability Assessment and Molecular Characterization of Oxeiptosis-Related OA Subtypes

**Figure 6:**
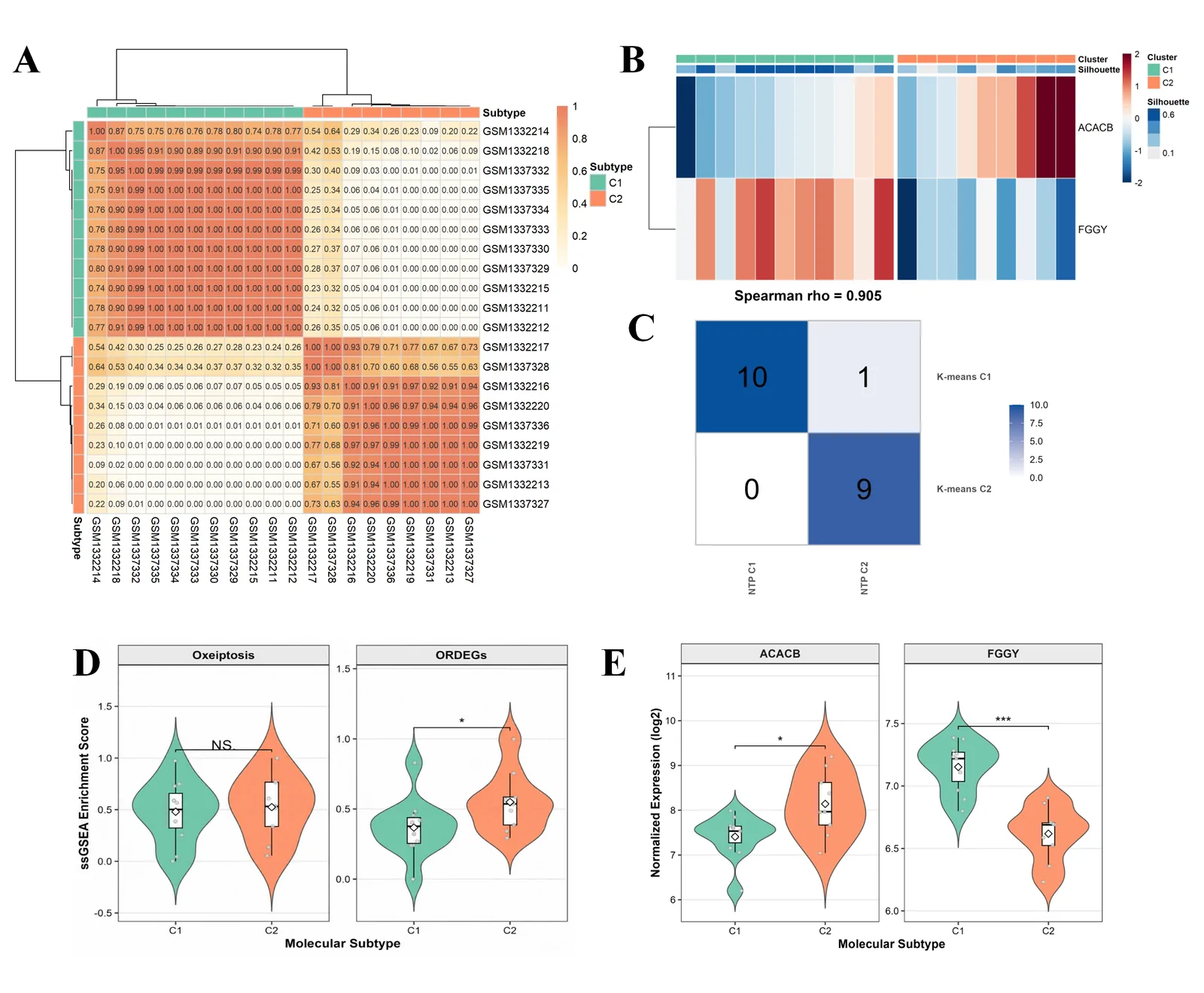
Internal consistency and molecular characteristics of oxeiptosis-associated OA molecular clusters. (**A**) Consensus clustering heatmap showing within-cluster similarity and separation of OA samples; (**B**) Expression heatmap of clustering features (*ACACB* and *FGGY*) across C1 and C2 clusters; (**C**) Comparison of consensus k-means and NTP subtype assignments; (**D**) ssGSEA analysis of oxeiptosis-related and ORDEG scores between clusters; (**E**) Expression patterns of *ACACB* and *FGGY* across the two clusters. OA, osteoarthritis; ssGSEA, single-sample gene set enrichment analysis; ORDEGs, oxeiptosis-related differentially expressed genes; NTP, nearest template prediction; CDF, cumulative distribution function. NS, not significant (≥0.05); * < 0.05; *** < 0.001.

The sample consensus matrix heatmap showed relatively high within-cluster consensus and separation between the two sample clusters (Figure 6A). All silhouette coefficients were positive, suggesting that the samples were internally consistent with their assigned clusters. Because the clustering was constructed exclusively from the standardized expression values of *ACACB* and *FGGY*, the corresponding expression heatmap was retained only to visualize the features defining the two clusters and was not considered independent evidence of biological differentiation (Figure 6B). NTP was additionally applied to the same 20 OA samples using the same *ACACB* and *FGGY* features. NTP and consensus k-means assignments were concordant in 19 of 20 samples, corresponding to an agreement rate of 95% and a Spearman correlation coefficient of 0.905 (Figure 6C). However, because both approaches were applied to the same samples and features, this analysis represents internal agreement between classification rules rather than independent validation or evidence of external reproducibility.

To explore phenotypic differences not directly used to define the clusters, ssGSEA was performed. No statistically significant difference was observed in the enrichment score based on the four canonical oxeiptosis regulators between the two clusters. The ORDEG enrichment score was higher in C2 in the original analysis; however, given the small sample size and the exploratory nature of the clustering, this finding was interpreted as hypothesis-generating and not as confirmation of a distinct biological subtype (Figure 6D). As *ACACB* and *FGGY* were the features used to construct the clusters, their between-cluster expression differences are mathematically expected. Therefore, the corresponding violin plots, if retained, are presented only as a descriptive visualization of the clustering features and not as independent validation of cluster-specific biomarkers (Figure 6E).

### 3.6. Independent External Validation of Oxeiptosis-Related Molecular Features

**Figure 7:**
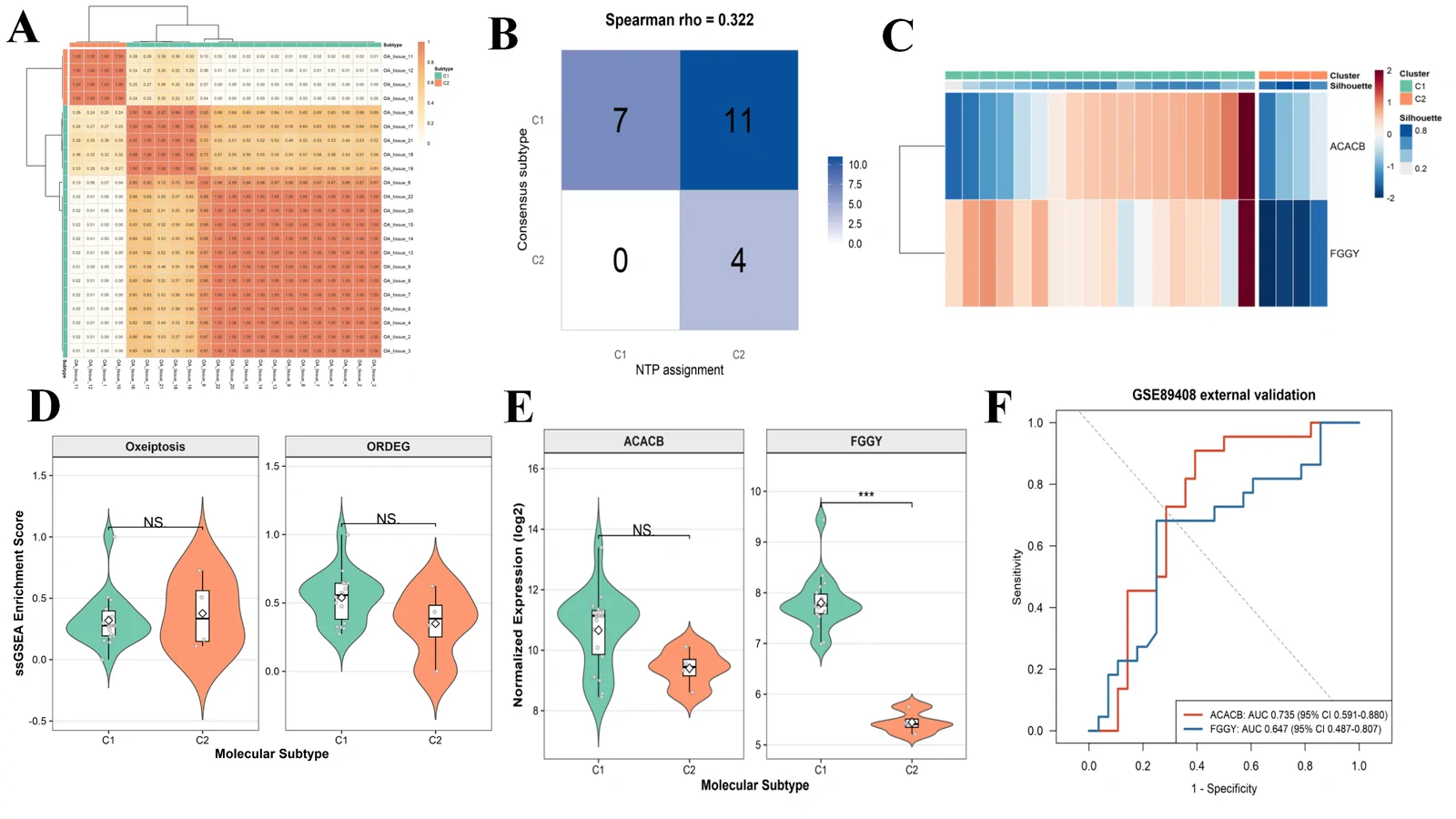
Independent external validation of the oxeiptosis-related molecular features in the GSE89408 cohort. (**A**) Consensus clustering heatmap based on *ACACB* and *FGGY* expression profiles in the external validation cohort, showing two potential molecular subtypes; (**B**) Comparison between consensus clustering and NTP classification using confusion matrix and correlation analysis; (**C**) Heatmap showing the expression patterns of *ACACB* and *FGGY* across the identified external molecular subtypes, together with cluster assignment and silhouette scores; (**D**) Comparison of oxeiptosis-related ssGSEA scores and ORDEG scores between C1 and C2 subtypes; (**E**) Differential expression analysis of *ACACB* and *FGGY* between C1 and C2 subtypes; (**F**) Receiver operating characteristic (ROC) curves evaluating the discriminatory performance of *ACACB* and *FGGY* for distinguishing OA patients from normal synovial tissues in GSE89408. OA, osteoarthritis; NTP, nearest template prediction; ssGSEA, single-sample gene set enrichment analysis; ORDEG(s), oxeiptosis-related differentially expressed gene(s); ROC, receiver operating characteristic; AUC, area under the curve. NS, not significant (≥0.05); *** < 0.001.

To further assess the reproducibility of the oxeiptosis-related molecular features identified in this study, we used an independent external cohort, GSE89408, which was not involved in candidate gene screening, machine learning modeling, or subtype construction. Based on *ACACB* and *FGGY* expression patterns, exploratory molecular typing was performed on the OA samples of this cohort. The results showed that two potential molecular subtypes (C1 and C2) could still be identified, with C1 containing 18 samples and C2 containing 4 samples, with a silhouette coefficient of 0.551, indicating a certain degree of separation between the two clusters in the independent cohort (Figure 7A–C). NTP analysis showed a positive correlation with the consensus clustering results (Spearman ρ = 0.322), providing limited support for the reproducibility of the molecular classification framework constructed based on oxeiptosis-related features (Figure 7B).

Further analysis of core gene expression patterns in the external cohort revealed a significant difference in *FGGY* between the C1 and C2 subtypes (*p* = 0.00251), with a continued high expression trend in the C1 subtype. While *ACACB* showed an increasing trend in the C2 subtype, this did not reach statistical significance (*p* = 0.0969) (Figure 7C,E). Furthermore, no significant differences were observed in oxeiptosis scores or ORDEG scores between the two subtypes (Figure 7D). In the evaluation of discriminatory performance, *ACACB* and *FGGY* achieved AUC values of 0.735 (95% CI: 0.591–0.880) and 0.647 (95% CI: 0.487–0.807), respectively, in distinguishing OA from normal synovial tissue (Figure 7F). In summary, the external validation results provided partial support for the potential value of *ACACB* and *FGGY* as oxeiptosis-related molecular features in OA heterogeneity analysis. However, due to the limited sample size of the external cohort, larger independent cohorts are needed to further evaluate their stability.

### 3.7. Immune Microenvironment Characteristics Between the Two Molecular Subtypes

**Figure 8:**
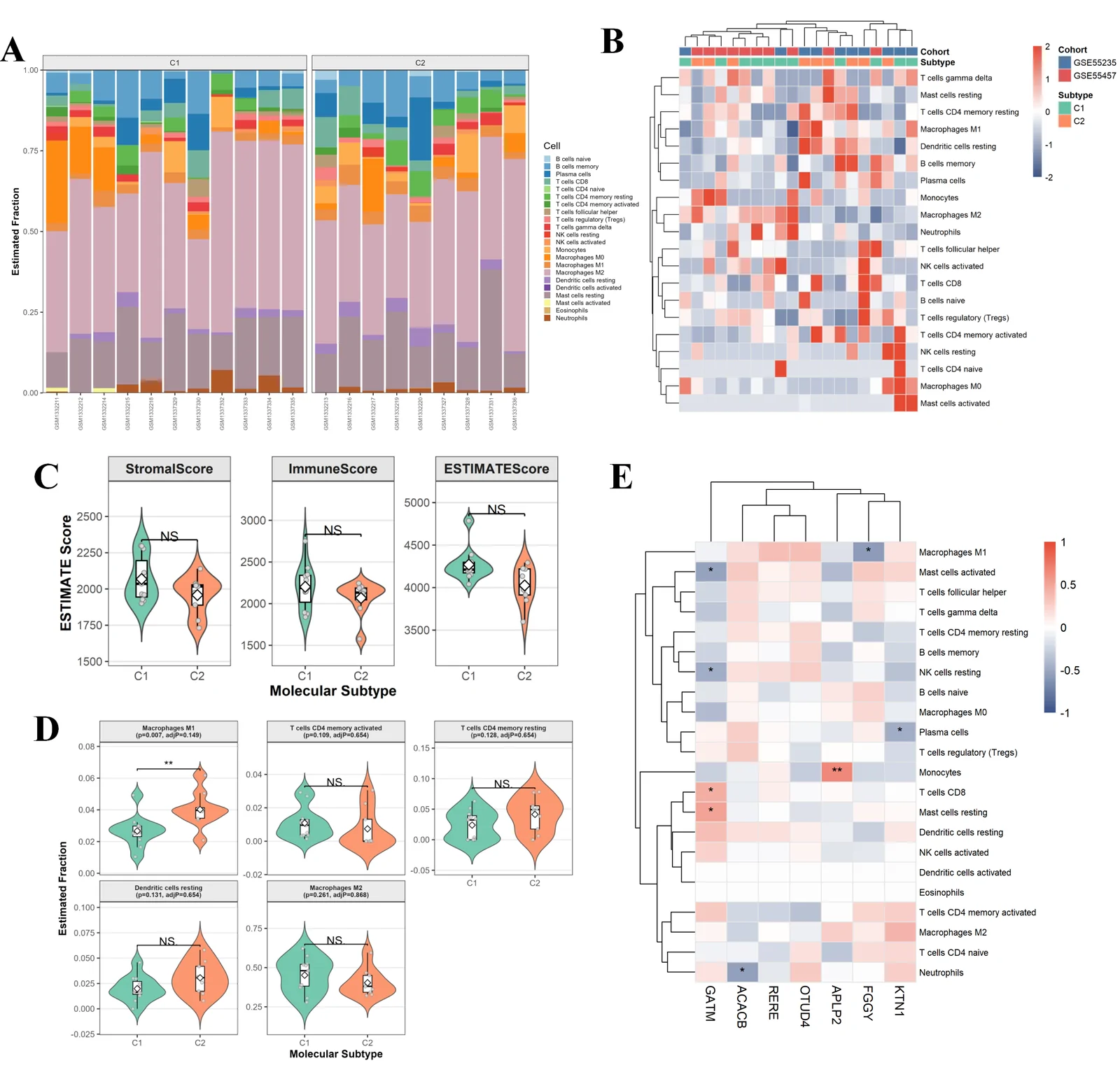
Immuneinfiltration characteristics of OA molecular subtypes and correlations between key genes and immune cells. (**A**) Stacked bar plot showing the proportions of infiltrating immune cell populations in the C1 and C2 subtypes; (**B**) Heatmap showing the correlation patterns among all infiltrating immune cell populations; (**C**) Violin plots showing the Stromal Score, Immune Score, and ESTIMATE Score between the C1 and C2 subtypes; (**D**) Violin plots showing the abundance distributions of differentially infiltrated immune cell populations; (**E**) Correlation heatmap showing the associations between seven oxeiptosis-related key genes and immune cell infiltration levels. Differences in immune cell infiltration between the C1 and C2 subtypes were assessed using the Wilcoxon rank-sum test, and multiple testing correction was performed using the Benjamini–Hochberg method. BH-FDR < 0.05 was considered statistically significant. Correlations between gene expression levels and immune cell infiltration were evaluated using Spearman correlation analysis. OA, osteoarthritis; ESTIMATE, Estimation of STromal and Immune cells in MAlignant Tumors using Expression data; CIBERSORT, Cell-type Identification By Estimating Relative Subsets Of RNA Transcripts; BH, Benjamini–Hochberg; FDR, false discovery rate; DEG(s), differentially expressed gene(s). NS, not significant (≥0.05); * < 0.05; *** < 0.001.

The CIBERSORT algorithm was applied to quantify the relative abundance of 22 immune cell types in OA synovial samples based on transcriptomic data. A stacked bar chart illustrated the overall immune cell composition across all samples (Figure 8A), showing comparable immune cell composition patterns between the two subtypes. The results demonstrated that the infiltration level of M1 macrophages was significantly higher in the C2 subtype than in the C1 subtype (BH-FDR < 0.01). No statistically significant differences were observed between the two subtypes in active CD4 memory T cells, resting CD4 memory T cells, resting dendritic cells, or M2 macrophages (Figure 8D). ESTIMATE analysis showed that neither the Stromal Score nor the Immune Score differed significantly between the C1 and C2 sample clusters. Although the median ESTIMATE Score was higher in C1 than in C2, the unadjusted *p* value was 0.0251, and the difference did not remain statistically significant after Benjamini–Hochberg correction (BH-FDR = 0.0752). Therefore, none of the three ESTIMATE-related scores showed statistically significant differences between the two sample clusters after multiple testing correction. Considering the limited sample size, these findings should be interpreted as exploratory observations (Figure 8C; Supplementary File S1).

The immune cell correlation heatmap demonstrated the correlation patterns among immune cell infiltration levels across the dataset and between subtypes (Figure 8B). Further correlation analysis between the seven oxeiptosis-related genes and immune cell infiltration revealed that *ACACB* was significantly negatively correlated with neutrophils (BH-FDR < 0.05), whereas *FGGY* showed a significant negative correlation with M1 macrophages (BH-FDR < 0.05). The remaining genes, including *GATM* and *APLP2*, also exhibited significant associations with various immune cell populations, including monocytes, NK cells, and CD8^+^ T cells (Figure 8E). These results suggest that *ACACB* and *FGGY*, as molecular features distinguishing the two oxeiptosis-related molecular subtypes, are associated with differences in inflammation-related immune cell infiltration, including M1 macrophages and neutrophils. Furthermore, the overall set of oxeiptosis-related genes exhibited extensive associations with immune cell infiltration patterns in the synovial microenvironment, providing potential molecular insights into the immune microenvironment heterogeneity between the C1 and C2 subtypes.

### 3.8. Identification of Gene Modules Associated with OA Molecular Subtypes Using WGCNA Analysis

**Figure 9:**
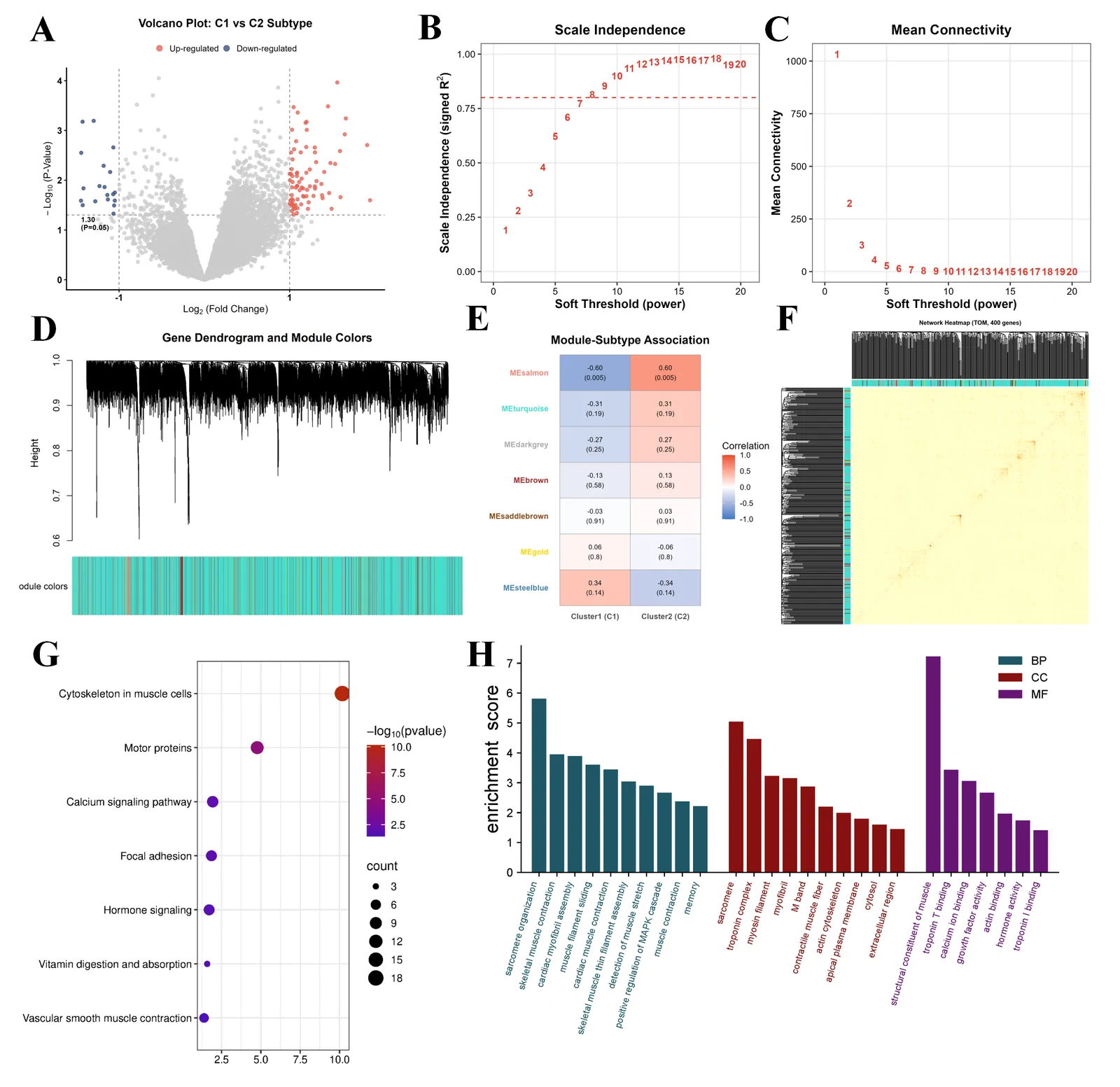
Construction of the WGCNA co-expression network and functional enrichment analysis of modules. (**A**) Volcano plots of differentially expressed genes between subtypes C1 and C2; (**B**) WGCNA soft-threshold scale independence curve; (**C**) WGCNA soft-threshold average connectivity curve; (**D**) Gene clustering tree and module color-coded map; (**E**) Correlation matrices between each gene module and the two molecular subtypes; (**F**) Heatmap of gene expression in modules; (**G**) KEGG pathway enrichment bubble plot of salmon module genes; (**H**) GO functional enrichment bar plot of salmon module genes.

Differential expression analysis was performed between the two molecular subtypes, and a volcano plot was generated (Figure 9A). A total of 99 significantly differentially expressed genes were identified, including 80 upregulated genes and 19 downregulated genes. By evaluating the scale-free topology fit index (R^2^) and average connectivity trends across soft-thresholding powers ranging from 1 to 20, a soft threshold of 7 was selected as the optimal parameter for subsequent WGCNA analysis (R^2^ ≥ 0.8) (Figure 9B,C). Based on this parameter, hierarchical clustering analysis identified seven gene modules (Figure 9D).

Module–trait correlation analysis revealed that the salmon module was significantly positively correlated with the C2 subtype (r = 0.60, *p* = 0.005), whereas the correlations between the remaining modules and the two subtypes were relatively weak (Figure 9E). Gene expression heatmaps of each module illustrated the overall expression patterns of module genes across samples, showing consistent expression trends among genes within the same module (Figure 9F). Genes within the salmon module, which exhibited a strong association with the C2 subtype, were further subjected to GO and KEGG enrichment analyses. KEGG pathway enrichment analysis demonstrated that the module genes were significantly enriched in pathways related to cytoskeletal regulation, motor proteins, calcium signaling, focal adhesion, and vascular smooth muscle contraction (Figure 9G). GO enrichment analysis showed that genes in this module were primarily enriched in biological processes related to cytoskeletal organization, skeletal muscle contraction, and myofibril structure formation. Enriched cellular components included troponin complexes, myofibrils, and contractile cytoskeletal structures, while molecular functions were mainly associated with calcium ion binding, actin binding, and growth factor binding (Figure 9H).

Based on the above functional enrichment and correlation analyses, the salmon module may represent a co-expression module associated with the C2 subtype. The hub genes within this module are mainly involved in biological processes related to cytoskeletal remodeling, calcium signaling, and cell adhesion, and may provide potential insights into the pathological phenotypic heterogeneity between the two oxeiptosis-related molecular subtypes of osteoarthritis.

## 4. Discussion

OA has long been regarded as a disease driven by factors such as abnormal mechanical loading and age-related degeneration. However, increasing evidence suggests that OA is not merely a process of structural wear and tear, but rather a systemic disease involving metabolic reprogramming, oxidative stress accumulation, chronic low-grade inflammation, and disruption of the immune microenvironment [19,20]. Particularly in OA-affected synovial tissue, ROS not only directly induce cellular damage but also function as signaling molecules that activate inflammatory pathways, including NF-κB and MAPK signaling, thereby regulating inflammatory factors and extracellular matrix metabolism [21]. Therefore, investigating cellular regulatory mechanisms under oxidative stress conditions is of great significance for elucidating the molecular heterogeneity underlying OA onset and progression.

This study provides the first systematic analysis of OA synovial molecular heterogeneity from the perspective of oxeiptosis. By integrating two independent synovial cohorts (GSE55235 and GSE55457), we found that OA-related differentially expressed genes were significantly enriched in MAPK, PI3K-AKT, FoxO, and NF-κB-related signaling pathways, and were closely involved in biological processes such as cellular response to hydrogen peroxide and oxidative stress. These findings suggest that ROS-related regulation may represent an important factor contributing to pathological alterations in OA synovium, which is consistent with previous studies [21]. However, unlike previous studies mainly focusing on the direct damage caused by ROS, this study further explored how cells sense and adapt to oxidative stress, suggesting that oxeiptosis-related molecular features may serve as important molecular links connecting oxidative stress, immune regulation, and alterations in the tissue microenvironment.

Oxeiptosis, as a ROS-induced, caspase-independent form of programmed cell death [11], differs from ferroptosis, which depends on iron-mediated lipid peroxidation [22], and pyroptosis, which induces cell lysis through inflammasome activation [23]. Oxeiptosis is characterized by a relatively mild and non-inflammatory form of cell death and may play an important role in maintaining tissue homeostasis under conditions of oxidative damage. Previous studies have suggested that oxeiptosis is not merely a form of cell death but may function as a protective mechanism that limits the further amplification of ROS-induced inflammation [24]. Therefore, under the chronic oxidative stress environment of OA, alterations in oxeiptosis status may reflect differences in the adaptive capacity of synovial cells in response to ROS stimulation, thereby contributing to the phenotypic heterogeneity observed among OA patients.

Through the integration of multiple machine learning algorithms, two key oxeiptosis-related molecular features, *ACACB* and *FGGY*, were further identified from seven ORDEGs, including *OTUD4*, *GATM*, *KTN1*, *FGGY*, *ACACB*, *RERE*, and *APLP2*. Notably, these two genes are associated with lipid metabolism and glucose metabolism, respectively, suggesting that altered oxeiptosis in OA may not represent an isolated biological event but may be closely linked to metabolic reprogramming and the maintenance of redox homeostasis.

Ma et al. reported that *KEAP1*-related redox signaling may influence oxidative stress responses and cell survival in OA, potentially modulating OA pathological processes characterized by oxidative stress and inflammation, suggesting that this pathway may contribute to the maintenance of joint tissue homeostasis [25]. *ACACB*, also known as acetyl-CoA carboxylase β, is a key enzyme involved in fatty acid metabolism and serves as an important regulatory node in mitochondrial energy metabolism [26]. Since metabolic disorders in lipid metabolism can promote ROS production and oxidative damage [27], and excessive ROS accumulation represents an important factor involved in oxidative stress-related cell death processes such as oxeiptosis [28]. Building upon these findings, our study observed that *ACACB* showed significant transcriptional associations with *KEAP1*-related redox genes, suggesting that the metabolic state represented by *ACACB* may be potentially linked to oxidative stress-related molecular programs and the oxeiptosis-related molecular phenotype in OA. Meanwhile, previous studies have shown that *ACACB* expression is downregulated in OA synovial tissues compared with normal tissues [29], further supporting its potential involvement in OA-related metabolic homeostasis imbalance. In the independent external validation cohort GSE89408, *ACACB* retained potential discriminatory ability between OA and normal synovial tissues (AUC = 0.735); however, its expression difference between external molecular subtypes did not reach statistical significance, suggesting that the contribution of *ACACB* may be influenced by sample heterogeneity and cohort size, and its robustness requires further validation.

On the other hand, *FGGY* belongs to the carbohydrate kinase family and was initially identified as a gene associated with amyotrophic lateral sclerosis [30]. Recent studies have mainly focused on its roles in cell proliferation and metabolic regulation, suggesting that *FGGY* may influence cell survival and apoptosis through multiple molecular pathways [31]. As a regulator associated with glucose metabolism, altered *FGGY* expression may reflect adaptive changes in cellular energy metabolism under oxidative stress conditions in OA. Glucose metabolism not only determines cellular energy supply but also affects mitochondrial oxidative metabolism and ROS production efficiency [32]. Therefore, the association between *FGGY* and oxeiptosis-related molecules, including *KEAP1* and *PGAM5*, suggests that glucose metabolic reprogramming may influence ROS-responsive processes through modulation of cellular redox status. Collectively, *ACACB* and *FGGY* may represent distinct metabolic adaptation patterns in OA and provide potential insights into the molecular heterogeneity associated with oxeiptosis.

Based on oxeiptosis-related molecular features, this study identified two OA molecular clusters (C1 and C2) with distinct transcriptional characteristics using an unsupervised clustering approach, suggesting the potential existence of different redox homeostasis regulatory patterns and immune microenvironment features among OA patients. Internal consistency analysis demonstrated good clustering stability of this classification in the discovery cohort. Furthermore, exploratory validation in the independent GSE89408 cohort revealed a similar trend of molecular separation; however, due to the limited sample size of the external cohort, this classification requires further validation in larger-scale cohorts. Notably, the C1/C2 classification does not simply reflect differences in the expression levels of individual genes, but may represent comprehensive biological variations among OA patients involving metabolic regulation, ROS response capacity, and inflammatory activation status. The C2 subtype was characterized by higher *ACACB* expression, elevated ORDEG scores, and increased infiltration of M1-type macrophages, suggesting that this subtype may exhibit more pronounced oxidative stress-related and inflammatory molecular features. As classical pro-inflammatory immune cells, M1-type macrophages secrete abundant pro-inflammatory cytokines, matrix metalloproteinases, and ROS, which may further amplify synovial inflammation and contribute to extracellular matrix degradation [33]. Meanwhile, the increased ESTIMATE composite score suggests that the C2 subtype exhibits more pronounced alterations in the tissue microenvironment. Considering the biological function of *ACACB* in lipid metabolism regulation, these findings suggest that the C2 subtype may represent an OA molecular phenotype characterized by the combined influence of abnormal lipid metabolism, enhanced oxidative stress responses, and immune activation.

In contrast, the C1 subtype showed predominant *FGGY* expression, which may represent another metabolic regulatory pattern. As a metabolism-related regulatory factor, *FGGY* may reflect cellular adaptation strategies for maintaining energy supply and metabolic homeostasis under oxidative stress conditions. Compared with the more pronounced immune activation state observed in the C2 subtype, the *FGGY*-high C1 subtype may exhibit distinct redox regulatory characteristics. To the best of our knowledge, this study is the first to incorporate *FGGY* into oxeiptosis-related molecular research in osteoarthritis and demonstrates its potential discriminatory ability in OA molecular subtype classification. In the independent GSE89408 validation cohort, *FGGY* remained highly expressed in the C1 subtype and showed a significant difference between the two molecular clusters (*p* = 0.00251), suggesting that *FGGY* may serve as a candidate molecular feature reflecting oxidative metabolic heterogeneity within OA. Notably, the significance of *FGGY* in OA disease discrimination and molecular subtype classification may differ. In the independent GSE89408 cohort, *FGGY* showed limited ability to distinguish OA from normal synovial tissues (AUC = 0.647); however, it maintained significant differential expression between the two oxeiptosis-related molecular subtypes, suggesting that *FGGY* may not represent a conventional diagnostic biomarker for OA, but rather a feature reflecting distinct oxidative metabolic states and molecular heterogeneity within OA. Given that OA is a complex disease with substantial molecular and clinical heterogeneity, a single molecular indicator may be insufficient to accurately classify all OA patients, but may contribute to identifying patient subgroups with different oxidative stress states, immune characteristics, or potential therapeutic response patterns. Therefore, the potential clinical relevance of *FGGY* may lie more in molecular stratification and identification of patient heterogeneity rather than disease diagnosis. However, the specific function of *FGGY* in OA and whether it directly participates in the oxeiptosis process remain to be experimentally validated. Future studies combining cellular and animal models are needed to further elucidate its underlying mechanisms.

WGCNA further revealed that the salmon module was significantly positively correlated with the C2 subtype, while the core oxeiptosis-related candidate gene *ACACB* showed an upregulated trend in the C2 subtype, suggesting that this module may be associated with oxidative metabolic dysregulation and inflammatory activation states related to the C2 subtype. Functional enrichment analysis demonstrated that genes within this module were significantly enriched in biological processes related to calcium signaling, cytoskeletal remodeling, focal adhesion, and vascular smooth muscle contraction. Among these processes, alterations in cellular dynamics and calcium homeostasis have been reported to influence mitochondrial function; disruption of calcium homeostasis may promote mitochondrial dysfunction and ROS production, thereby affecting cellular responses associated with oxidative stress and potentially influencing oxeiptosis-related cellular states [34]. Meanwhile, abnormalities in cell adhesion may disrupt mechanical and signaling interactions between synovial cells and the extracellular matrix (ECM), thereby contributing to the persistence of synovial inflammation [35]. Therefore, in combination with the above characteristics of the C2 subtype, the salmon module may provide potential insights into the cellular structural, metabolic, immune activation, and tissue remodeling features of the C2 subtype. These findings further support the concept that oxeiptosis-related molecular signatures do not represent isolated cell death events but may be embedded within a complex regulatory network involved in remodeling the OA synovial microenvironment, providing a foundation for further investigation into the mechanisms underlying OA molecular subtype formation.

Based on the oxeiptosis-associated molecular characteristics identified by *ACACB* and *FGGY*, this study further revealed potential differences in redox homeostasis and molecular heterogeneity among OA patients, providing a new basis for stratifying the molecular states of different OA subgroups. Traditional OA therapeutic strategies primarily focus on symptom relief and structural preservation, with limited consideration of molecular heterogeneity among patients. This study suggests that OA samples corresponding to the C2 subtype, characterized by M1 macrophage enrichment and enhanced oxidative stress-related features, may represent an inflammation-activated molecular phenotype, whereas other subtypes with distinct metabolic characteristics may exhibit different potential therapeutic considerations. Therefore, stratification based on oxeiptosis-related molecular features may provide a theoretical foundation for exploring more precise OA intervention strategies in the future. However, it should be emphasized that these interpretations are primarily derived from bioinformatics analyses and require further experimental validation to determine their potential clinical relevance.

This study has several limitations. Although multiple public transcriptomic cohorts were integrated, and multi-algorithm feature selection, patient-level cross-validation, repeated resampling, and independent external validation were employed to improve the robustness of the findings, the sample sizes of the individual cohorts were still relatively limited. Therefore, the stability of *ACACB* and *FGGY* and their value for molecular stratification require further validation in larger, independent cohorts with complete clinical information. In addition, GEO datasets mainly provide transcriptional-level information, and thus the present study could not fully capture protein expression, cell type-specific characteristics, or the dynamic changes in oxeiptosis. Transcriptional associations alone are also insufficient to establish a causal relationship between the oxeiptosis-related molecular features and OA development and progression, and whether these genes directly participate in the development of oxeiptosis remains to be experimentally validated. This study used CIBERSORT combined with the LM22 feature matrix to infer immune cell composition, but this method has certain limitations in the application of transcriptome analysis in complex synovial tissues. Therefore, the immune cell abundance in this study should be considered as a calculated and inferred immune-related feature, rather than a direct representation of actual immune cell infiltration or macrophage polarization. Further confirmation through single-cell sequencing and experimental validation is needed in the future.

Second, clinical and pathological information in the publicly available GEO cohorts was incomplete and heterogeneous to varying degrees, making consistent and reliable adjustment or stratification for clinical covariates across cohorts difficult. Therefore, the C1 and C2 subtypes defined in this study should be regarded as exploratory oxeiptosis-related molecular patterns identified at the transcriptional level rather than clinically validated patient subtypes. Although GSE89408 provided independent external validation, its sample size was still limited. Therefore, these results were mainly used to assess the exploratory reproducibility of the molecular features rather than to provide definitive validation of the subtypes. In addition, potential confounding factors, including disease severity, treatment history, comorbidities, tissue source, and demographic characteristics, could not be consistently adjusted across cohorts, further limiting the clinical generalizability of the findings. Finally, future studies integrating single-cell sequencing, proteomics, and in vivo and in vitro experimental models may further validate the roles of *ACACB*, *FGGY*, and related molecular networks in OA at the cellular and functional levels. Gene intervention models targeting *ACACB* or *FGGY*, combined with ROS measurement, assessment of oxidative stress status, mitochondrial function evaluation, and validation of the *KEAP1*-*PGAM5-AIFM1* signaling axis, may help further determine whether these molecular features directly participate in oxeiptosis-related biological processes. Such studies will help determine whether the transcriptional associations identified in the present study can be translated into experimentally verifiable functional mechanisms and further clarify their potential clinical significance.

## 5. Conclusions

This study established an OA molecular subtyping framework based on oxeiptosis-related molecular features. By integrating differential expression analysis, machine learning, unsupervised clustering, immune infiltration analysis, WGCNA, and external validation, we systematically investigated the relationship between oxeiptosis and molecular heterogeneity in OA. *ACACB* and *FGGY* were identified as two potential oxeiptosis-related molecular features, and further analyses suggested an association between oxeiptosis-related molecular features and OA subtypes with distinct immune microenvironment characteristics. Independent external validation further provided partial support for the reproducibility of these molecular patterns, particularly the *FGGY*-related features. This study provides a new perspective on the potential role of oxeiptosis-related regulation in OA heterogeneity and provides a basis for future molecular stratification studies in OA.

## Figures and Tables

**Table 1:** Summarizes the characteristics and analytical purposes of the GEO transcriptomic datasets used in this study.

Data Number	Platform Information	Osteoarthritis Group	Comparison Group	Species	Research Purposes
GSE55235	GPL96	10	10	Homo sapiens	DEG screening; exploratory clustering analysis
GSE55457	GPL96	10	10	Homo sapiens	DEG screening; exploratory clustering analysis
GSE206848	GPL570	7	7	Homo sapiens	Oxeiptosis-related screening
GSE46750	GPL10558	12	12	Homo sapiens	Machine learning filtering
GSE89408	GPL11154	22	28	Homo sapiens	External validation of candidate genes; exploratory repeated clustering of OA samples

GSE55235 and GSE55457 were used as discovery cohorts for identifying OA-related DEGs. GSE206848 was used to identify genes associated with the oxeiptosis-related expression signature through correlation analysis with canonical oxeiptosis regulators. GSE46750 was used as the feature-selection cohort for machine learning-based identification of key molecular features. GSE89408 was used as an independent external validation cohort to evaluate the reproducibility of the identified molecular features and molecular patterns. OA, osteoarthritis; GEO, Gene Expression Omnibus; DEG(s), differentially expressed gene(s).

## Data Availability

The datasets GSE55235, GSE55457, GSE206848, and GSE46750 analyzed in this study are openly available in the Gene Expression Omnibus (GEO) database at https://www.ncbi.nlm.nih.gov/geo/ (accessed on 18 July 2026), with the accession numbers GSE55235, GSE55457, GSE206848, and GSE46750.

## Supplementary Materials

The following supporting information can be downloaded at https://www.mdpi.com/article/10.3390/biomedicines14092079/s1.
