# Supplementary material for: Oxeiptosis-Associated Molecular Subtyping and Immune Microenvironment Heterogeneity in Osteoarthritis
Source: Biomedicines. 2026 Sep 16;14(9):2079. doi: 10.3390/biomedicines14092079 (PMC13604199; doi:10.3390/biomedicines14092079)
Supplement: Supplementary file 1 [file biomedicines-14-02079-s001.zip › Figure S1.pdf]

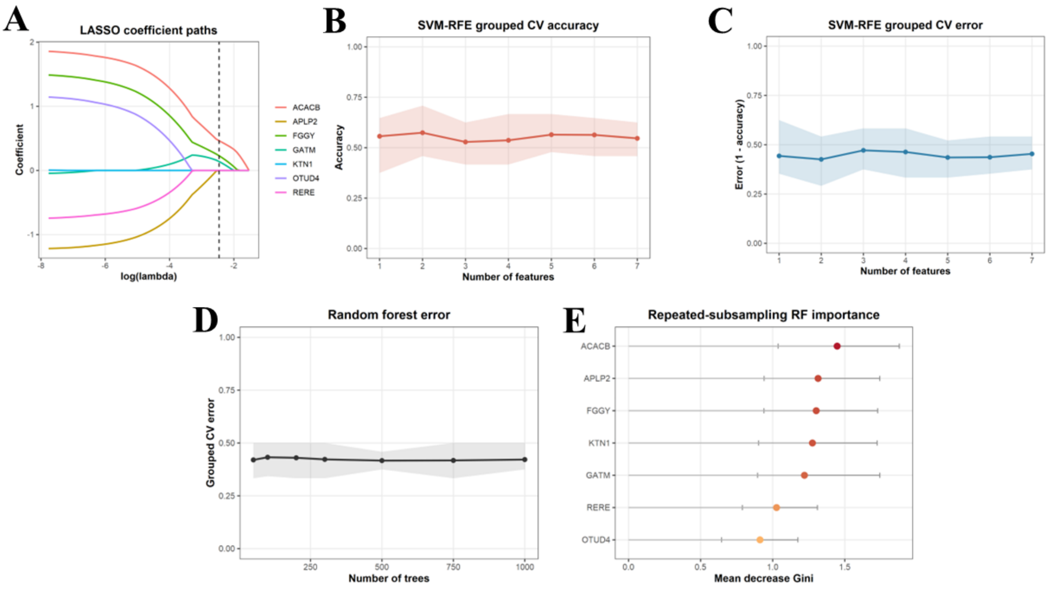

Supplementary Figure S1 Model optimization and feature importance analyses for machine learning-based selection of oxeiptosis-related candidate genes.

(A) LASSO coefficient path plot showing the changes in regression coefficients of the seven putative oxeiptosis-related differentially expressed genes across different values of log(λ). (B-C) Internal cross-validation performance of the SVM-RFE model, showing grouped cross-validation accuracy (B) and cross-validation error (C) across different numbers of selected features. (D) Random forest model error curve showing the relationship between the number of decision trees and prediction error. (E) Repeated patient-level resampling analysis of random forest feature importance based on Mean Decrease Gini, showing the relative contribution of each candidate gene. LASSO, least absolute shrinkage and selection operator; SVM-RFE, support vector machine-recursive feature elimination; RF, random forest; λ , penalty parameter.
